# Heparanase Localization during Palatogenesis in Mice

**DOI:** 10.1155/2013/760236

**Published:** 2013-02-12

**Authors:** Azumi Hirata, Kentaro Katayama, Takehito Tsuji, Nagato Natsume, Toshio Sugahara, Yuichi Koga, Kazufumi Takano, Yoshinori Otsuki, Hiroaki Nakamura

**Affiliations:** ^1^Department of Anatomy and Cell Biology, Faculty of Medicine, Osaka Medical College, Takatsuki 569-8686, Japan; ^2^Laboratory of Veterinary Physiology, Nippon Veterinary and Life Science University, Musashino 180-8602, Japan; ^3^Graduate School of Environmental and Life Science, Okayama University, Okayama 700-8530, Japan; ^4^Division of Research and Treatment for Oral and Maxillofacial Congenital Anomalies, School of Dentistry, Aichi Gakuin University, Nagoya 464-0821, Japan; ^5^Departments of Material and Life Science, Graduate School of Engineering, Osaka University, Suita 565-0871, Japan; ^6^Laboratory of Biological Chemistry, Department of Biomolecular Chemistry, Kyoto Prefectural University, Kyoto 606-8522, Japan; ^7^Department of Oral Histology, Matsumoto Dental University, Shiojiri 399-0781, Japan

## Abstract

Palatogenesis is directed by epithelial-mesenchymal interactions and results partly from remodeling of the extracellular matrix (ECM) of the palatal shelves. Here, we assessed heparanase distribution in developing mouse palates. No heparanase was observed in the vertically oriented palatal shelves in early stages of palate formation. As palate formation progressed, the palatal shelves were reorganized and arranged horizontally above the tongue, and heparanase localized to the epithelial cells of these shelves. When the palatal bilateral shelves first made contact, the heparanase localized to epithelial cells at the tips of shelves. Later in fusing palatal shelves, the cells of the medial epithelial seam (MES) were labeled with intense heparanase signal. In contrast, the basement membrane heparan sulfate (HS) was scarcely observed in the palatal shelves in contact. Moreover, perlecan labeling was sparse in the basement membrane of the MES, on which laminin and type IV collagen were observed. Moreover, we assessed the distribution of matrix metalloproteinase- (MMP-) 9, MMP-2, and MMP-3 in developing mouse palates and these MMPs were observed in the MES. Our findings indicated that heparanase was important for palate formation because it mediated degradation of the ECM of palatal shelves. Heparanase may, in concert with other proteases, participate in the regression of the MES.

## 1. Introduction

Development of the mammalian secondary palate is a dramatic event that depends on multiple steps and a network of several factors. Palate formation starts with the appearance of two palatal shelves that protrude from the lateral walls of oronasal cavity. Both palatal shelves grow downward vertically along the side of the tongue. Subsequently, the palatal shelves are raised above the tongue, which moves downward as the mandible elongates. The bilateral palatal shelves grow towards each other until they make contact and adhere at the midline along the medial edge epithelium (MEE). Finally, the epithelium disappears from the shelves, thus allowing for complete palatal fusion [1].

The processes of the morphological changes and the accompanying histological changes in the palate are directed by epithelial-mesenchymal interactions [1]. They result partly from remodeling of the extracellular matrix (ECM) of the palatal shelves. Components of the ECM such as hyaluronan and fibronectin are expressed in the mesenchyme of the palatal shelves [2] and may contribute to the elevation of the palatal shelves [3]. After adhesion of the bilateral palatal shelves in the midline, formation and subsequent disappearance of the medial epithelial seam (MES) are essential for complete palatal fusion. Changes in the distribution of ECM components within the basement membrane of the MES (e.g., type IV collagen, laminin, and perlecan) during palate formation have been examined [4, 5]. However, the fate of the cells of the MES is controversial [2, 6].

Moreover, palatogenesis requires proteolytic degradation of the ECM, and some proteases, such as matrix metalloproteinases (MMPs), are responsible for remodeling of the ECM during palatal fusion [7]. MMPs mediate changes in the basement membrane (BM) components of the MES [8].  Heparanase, an endoglucuronidase, cleaves HS chain in perlecan [9–11], and heparanase releases ECM resident, HS-bound polypeptides, and then converts them to bioactive molecules. Heparan sulfate proteoglycan (HSPG) binds many ECM molecules, growth factors, and cell surface receptors via HS chains, and HSPG has multiple developmental and physiological functions [12]. Afterwards, heparanase homolog, termed heparanase 2 (Hpa2), was cloned [13]. It encodes three proteins generated by alternative splicing (Hpa2a, Hpa2b, Hpa2c) and shares an overall identity of ~40% with heparanase. Hpa2 tends to show tissue-specific patterns of expression. Additionally, Hpa2 exhibits no enzymatic activity typical of heparanase and Hpa2c protein inhibits heparanase enzymatic activity [14]. We have previously reported that heparanase, not Hpa2, secreted by the cells of Hertwig's epithelial root sheath may contribute to formation of tooth roots by degrading the dental basement membrane [15]. However, the role of heparanase during formation of the palate has not yet been reported. Mechanisms leading to the disappearance of the cells of the MES are controversial [2, 6]. The aim of the present study was to use immunohistochemistry (IHC) to determine heparanase localization in the developing murine palatal shelves to assess whether heparanase might be involved in palate formation. Moreover, we used IHC to assess the distribution of ECM components and MMPs in fusing palatal shelves to determine whether expression of these proteins correlated with the disappearance of the MES cells during palatogenesis.

## 2. Materials and Methods

All animal experiments were conducted in accordance with the Guidelines for Animal Experiments, Okayama University, Okayama, Japan.

### 2.1. Tissue Preparation for Histology

C57BL/6By mice were used in the present study. Pregnant females (*n* = 9) that had mated with males were euthanized with CO_2_ such that six embryos each could be harvested at different developmental stages, that is, embryonic day 13.5 (E13.5), E14.5, and E15.5. Twenty-seven embryos along with three newborn mice (P0) were immersed in 4% paraformaldehyde and 0.1% glutaraldehyde in 0.05 M phosphate buffer (pH 7.4) for heparanase, heparan sulfate, MMP-2, MMP-3, and MMP-9 IHC. The remnants of embryos and three newborn mice were immersed in an acid-alcohol fixative comprising 96% ethanol, 1% acetic acid, and 3% distilled water for perlecan, laminin, and type IV collagen IHC [15]. Heads were dissected, immersed in the same fixative for 20 h at 4°C, and decalcified in 5% EDTA, pH 7.4, for 2 days at 4°C.

For light or immunofluorescent microscopy, specimens were dehydrated in a graded ethanol series and embedded in paraffin. Sections (4 *μ*m thick) were prepared and dewaxed with xylene and graded ethanol.

### 2.2. Heparanase, Heparan Sulfate, and MMPs IHC

Serial sections were transferred to 5 mM periodic acid for 10 min to block endogenous peroxidase and then immersed in PBS containing 10% BSA for 30 min. For heparanase IHC, sections were washed in PBS and then incubated with an antiheparanase polyclonal antibody (3 *μ*g/mL) [15] for 12 h at 4°C. The heparanase rabbit polyclonal antibody was generated using a cysteine-conjugated peptide corresponding to residues 28–45 (DDVVDLFYTKRPLRSVS) of mouse heparanase (GenBank accession no. AY077467) [15, 16]. This antibody specifically reacts with an active form of mouse heparanase, not Hpa2 [15]. Sections were then incubated with a secondary antibody (ChemMate ENVISION; Dako Cytomation, Glostrup, Denmark) for 1 h at RT. For heparan sulfate IHC, sections were washed in PBS and then incubated with an anti-heparan sulfate (NAH46 epitope) monoclonal antibody (Seikagaku Biobusiness Co., Ltd.; Tokyo, Japan) diluted 1 : 1000 for 12 h at 4°C. They were reacted with Histofine MOUSESTAIN KIT (Nichirei Biosciences, Inc.; Tokyo, Japan) for 1 h at RT. For MMPs IHC, sections subsequently incubated with an anti-MMP-2 polyclonal antibody diluted 1 : 100 (MILLIPORE, Temecula, CA, USA) or an anti-MMP-3 polyclonal antibody diluted 1 : 100 (MILLIPORE) or an anti-MMP-9 polyclonal antibody diluted 1 : 100 (MILLIPORE) for 12 h at 4°C. Sections were then incubated with ChemMate ENVISION (Dako Cytomation) for 1 h at RT. Control sections were incubated with rabbit IgG preimmune serum, then incubated with a secondary antibody (ChemMate ENVISION or HISTOFINE) for 12 h at 4°C. Immunoreactivity was visualized using diaminobenzidine (DAB) (DAKO, Carpinteria, CA, USA). Sections were then counterstained with hematoxylin and examined under an All-in-one Type Fluorescence Microscope (BZ-9000; Keyence, Osaka, Japan) using BZ Analyzer software (BZ-9000; Keyence). These immunohistochemical staining procedures were performed in 120 serial sections from each mouse.

### 2.3. Localization of Perlecan, Laminin, and Type IV Collagen

Serial sections were transferred to 5 mM periodic acid for 10 min at room temperature (RT) to block endogenous peroxidase and were then immersed in PBS containing 10% BSA for 30 min. For double staining of perlecan and laminin, sections were treated with 15,000 U/mL of hyaluronidase (Sigma, St. Louis, MO, USA) in PBS for 30 min at 37°C. Sections were washed with PBS and then incubated with an anti-perlecan (clone A7L6) monoclonal antibody (MILLIPORE) diluted 1 : 200 and an anti-laminin polyclonal antibody (Sigma) diluted 1 : 100 for 12 h at 4°C. Clone A7L6 recognizes domain IV of the core protein of perlecan. Anti-laminin antibody was developed in rabbit using laminin purified from the basement membrane of Englebreth Holm-Swarm (EHS) mouse sarcoma as the immunogen. For double staining of perlecan and type IV collagen, sections were treated with 15,000 U/mL of hyaluronidase (Sigma) in PBS for 30 min at 37°C and 0.1% pepsin in 0.01 N HCl for 15 min at RT. Sections were washed in PBS and then incubated with an anti-perlecan monoclonal antibody diluted 1 : 200 (MILLIPORE) and an anti-type IV collagen polyclonal antibody (PROGEN Biotechnik; Heidelberg, Germany) diluted 1 : 100 for 12 h at 4°C. For immunofluorescence, sections were incubated with Alexa Fluor-488 goat anti-rat IgG (Molecular Probes, Eugene, OR) diluted 1 : 200 and Alexa Fluor-594 goat anti-rabbit IgG (Molecular Probes) diluted 1 : 200 for 1 h at RT. Sections were then observed under a microscope (Keyence). These immunohistochemical staining procedures were performed in 60 serial sections from each mouse.

## 3. Results

### 3.1. Localization of Heparanase and Heparan Sulfate

**Figure 1:**
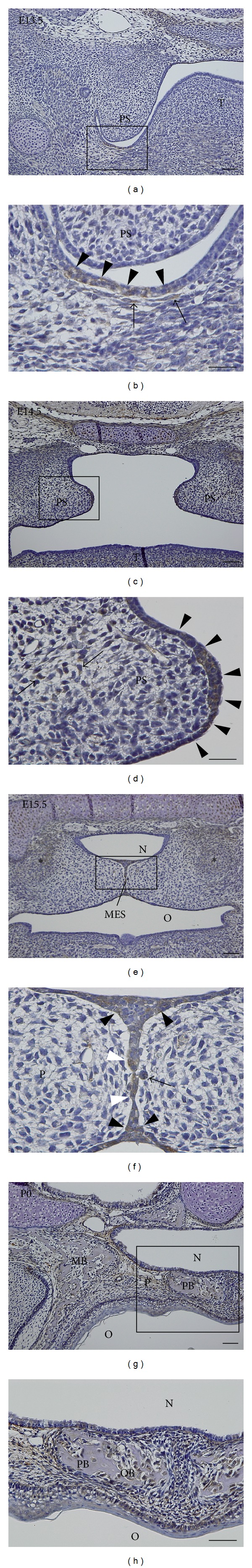
Light micrographs showing the localization of heparanase during palate formation. (a) Palatal shelf of a mouse embryo at E13.5. Heparanase signal was not evident in the palatal shelf. (b) Higher magnification of square marked in (a). Some epithelial cells and mesenchymal cells facing the palatal shelf at the bottom of the tongue had weak heparanase labeling (arrows and arrowheads). (c) Palatal shelves of a mouse at E14.5. The epithelial cells of palatal shelves had heparanase labeling. (d) Higher magnification of the square marked in (c). Intense reactivity was evident in the cytoplasm of the epithelial cells (arrowheads). Some stromal cells also had heparanase reactivity (arrows). (e) Palate of a mouse embryo at E15.5. The MES was observed in the middle of palate. Epithelial cells of palate and mesenchymal cells located at the ossification center (∗) had heparanase reactivity. (f) Higher magnification of the square marked in (e). The cells of the MES (white arrowheads), epithelial triangle (arrowheads), and epithelial island (arrow) show heparanase reactivity. (g) Palate of a mouse at P0. The palatal and maxillary bone was observed. (h) Higher magnification of the square marked in (g). Heparanase localization was evident in osteoblasts on the palatal bone surface. Strong reactivity of heparanase was evident in the basal cells of oral and nasal epithelium of the palate. Some stromal cells also had heparanase reactivity. PS, palatal shelf; P, palate; T:, tongue; MES, medial epithelial seam; PB, palatal bone; MB, maxillary bone; OB, osteoblasts; O, oropharynx; N, nasopharynx. Bars: (a, c, e, g, and h), 50 *μ*m; (b, d, f), 25 *μ*m.

**Figure 2:**
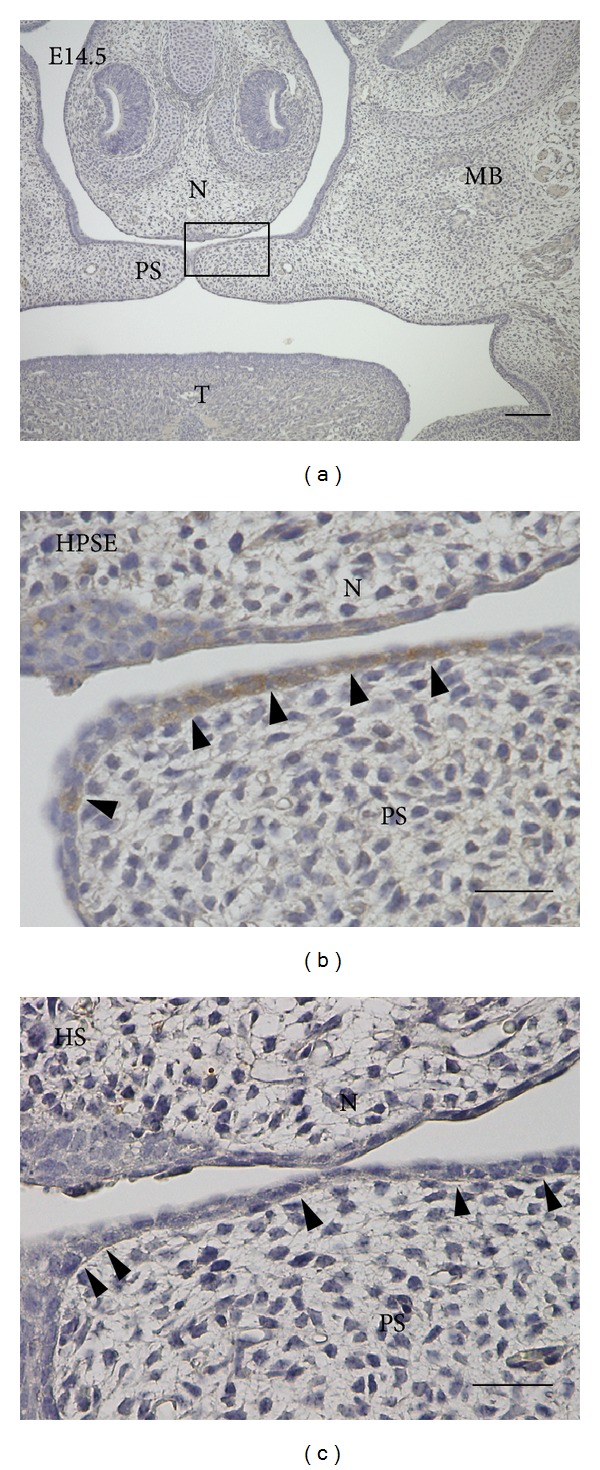
Light micrographs showing the localization of heparanase and heparan sulfate in palatal shelves of a mouse embryo at E14.5. (a) The palatal shelves were horizontal in apposition. (b) A higher magnification of the square marked in (a). Some epithelial cells at the tip of palatal shelves had heparanase labeling (arrowheads). Adjacent section to the section shown in (c). (c) HS labeling was diffuse and weak in the basement membrane at the tip of palate epithelium (arrowheads). Adjacent section to the section shown in (b). PS, palatal shelf; T, tongue; MB, maxillary bone; N; nasal septum. Bars: a, 100 *μ*m; b, c, 25 *μ*m.

At E13.5, the palatal shelves were positioned bilaterally along the sides of the tongue and elongated vertically and perpendicular to the tongue (Figure 1(a)). Heparanase signal was not observed in the palatal shelves at E13.5. Some epithelial cells and underlying mesenchymal cells facing the palatal shelves at the bottom of the tongue showed weak labeling (Figure 1(b)). By E14.5, the shelves had reoriented such that they had elongated horizontally above and parallel with the surface of the tongue. Intense heparanase immunoreactivity was evident in the cytoplasm of epithelial cells of the palatal shelves. Heparanase was also expressed at the mesenchyme (as shown by arrows) (Figures 1(c) and 1(d)). At E15.5, the bilateral palatal shelves had connected, and the MES was observed at the midline (Figure 1(e)). Cells of the epithelial triangle, the MES, and epithelial island had strong heparanase labeling (Figure 1(f)). As palatogenesis was complete, heparanase localization was evident in the basal cells of oral and nasal epithelium of the palate at P0 (Figures 1(g) and 1(h)). Osteoblasts on the palatal and maxillary bone surface also had heparanase reactivity (Figures 1(g) and 1(h)). Additionally, some epithelial cells located near the tip of the shelves that had made contact above the tongue had heparanase immunoreactivity (Figures 2(a) and 2(b)). In contrast, HS-labeling was faint in the basement membrane located nearby the tip of shelves (Figure 2(c)).

No specific immunoreactivity was observed in control sections (see Figure S1 in Supplementary Material available online at http://dx.doi.org/10.1155/2013/760236).

### 3.2. Distribution of Perlecan, Laminin, and Type IV Collagen in the MES

**Figure 3:**
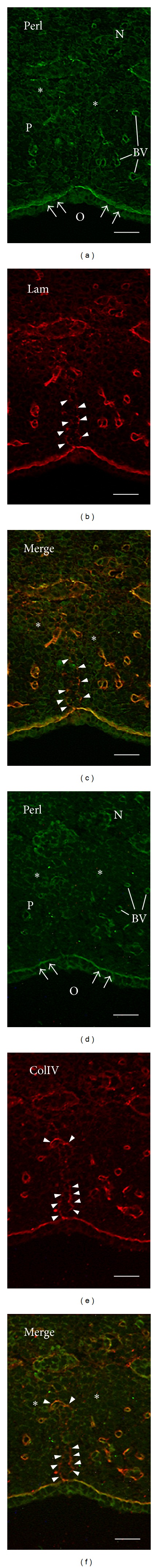
Micrographs of double-immunofluorescence staining reveal the localization of perlecan and laminin ((a)–(c)) or type IV collagen ((d)–(f)) in palate of a mouse embryo at E15.5. ((a), (d)) Perlecan reactivity was evident in the basement membrane of oral palate epithelium (arrows), blood vessels, and some stroma (∗) in palate. ((b), (e)) Weak or discontinuous laminin (b) or type IV collagen (e) immunoreactivity was evident in the basement membrane of the MES (arrowheads). ((c), (f)) Colocalization of perlecan and laminin (c) or type IV collagen (f) was observed in the basement membrane of oral epithelium and blood vessels. Some parts of the basement membrane of the MES had only laminin (c) or type IV collagen (f) reactivity (arrowheads). Adjacent sections. P, palate; O, oropharynx; N, nasal septum; BV, blood vessels; MES, medial epithelial seam. Bars: 25 *μ*m.

The MES was first observed at the midline of the connected palate at E15.5 (Figure 3(a)). ECM components were evident in the palate. Perlecan was diffusely distributed in stroma; moreover, perlecan, laminin, and type IV collagen were evident in the basement membrane of blood vessels (Figures 3(a), 3(b), 3(d), and 3(e)). Furthermore, perlecan labeling was largely absent on the basement membrane of the MES, which was labeled with laminin signal (Figures 3(a)–3(c)), or perlecan labeling on the basement membrane of the MES was partially absent, although type IV collagen labeling was visible (Figures 3(d)–3(f)).

No specific immunoreactivity was detected in the control sections (data not shown).

### 3.3. Localization of MMP-9, MMP-2, and MMP-3 in the MES

**Figure 4:**
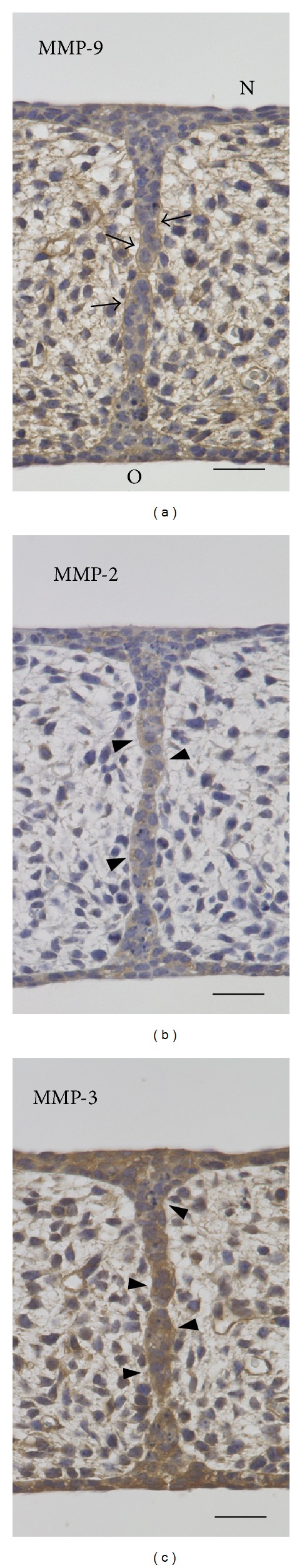
Light micrographs showing the localization of MMP-9 (a), MMP-2 (b), and MMP-3 (c) in the MES of palate of a mouse embryo at E15.5. (a) MMP-9 reactivity was observed throughout the basement membrane of the MES (arrows). Stroma cells had diffuse MMP-9 labeling. (b) Moderate MMP-2 reactivity was seen in cells of the MES (arrowheads). Stroma cells had weak labeling. (c) Strong labeling of MMP-3 was seen in cells of the MES (arrowheads) and stroma cells. P, palate; O, oropharynx; N, nasopharynx. Bars: 25 *μ*m.

Immunolocalizations of MMP-9, MMP-2, and MMP-3 in the MES at E15.5 are shown in Figure 4. MMP-9, MMP-2, and MMP-3 were evident in the MES. Intense MMP-9 signal was observed around the cells of the MES that corresponded to the basement membrane; moderate MMP-2 signal and strong MMP-3 signal were evident in the cells of the MES. MMP-2 and MMP-3 signal were evident in the mesenchyme of the palate.

No specific immunoreactivity was observed in the control sections incubated without any primary antibody (Supplemental data). 

## 4. Discussion

Here, we assessed the distribution of heparanase during palate formation in mice. In the initial stage of palate formation, heparanase signal was not evident in the palatal shelves. As palate formation progressed to elevation, heparanase signal was evident in the epithelial cells of palatal shelves. Heparanase signal was evident in some nasal epithelium cells of palate as these shelves made contact. At this same stage, the basement membrane HS was faint and largely absent from the epithelial cells near the tip of shelves. After bilateral palatal shelves connected, heparanase signal in cells of the MES was very strong. These results suggest that epithelial cells of the palatal shelves mediated the degradation of basement membrane by secreting heparanase during palate formation. These data also suggest that heparanase activity and the disappearance of basement membrane may have been spatially and temporally coordinated. Heparanase secreted by the palatal epithelial cells may participate in the formation of the palate, particularly in the fusion of palatal shelves via the degradation of palate basement membrane. Heparanase might also be required for the cleavage of HS on the epithelial plasma membrane during palate connection.

Moreover, heparanase labeling was evident in osteoblasts that faced the surface of the palatal bone and the maxillary bone. Increases in HS signal were evident in the bone matrix as palate formation progressed (data not shown). The presence of HS in bone and its association with skeletal physiologic and pathologic processes are well established [17–19]; osteoblasts and osteoclast lineage cells synthesize HSPG and this HSPG localized on their plasma membrane is involved in the binding of cell-cell interaction between osteoblasts and osteoclast lineage cells. Moreover, HSPG in bone matrix is involved in cell-matrix attachment and is also a reservoir of bioactive molecules and concerned with bone metabolism [20, 21]. Additionally, heparanase expression in osteoblasts and its biological function in osteogenesis have been documented [22–24]. Our data also provided evidence that heparanase localized in osteoblasts. Perlecan expression is associated with increased lacunocanalicular space in cortical bone [25]. This data is consistent with an inhibitory role for perlecan heparan sulfate chains in biomineralization and with the current study which shows that heparanase signal is associated with increased osteogenesis [25]. Heparanase may have an important role during the process of bone formation in palatogenesis.

Furthermore, heparanase expression is not restricted to pathological conditions and the activity has been found in hair follicle [26, 27] and in the skin [28]. Heparanase was detected in the outer root sheath of murine hair follicle [26], while it mainly expressed in the inner root sheath of human hair follicle [27], suggesting, despite of differential expression between the species, that heparanase may be a key factor in differentiation of a follicular stem cell and hair homeostasis. In human epidermis, heparanase expression has been reported to be closely related to keratinocyte differentiation and was mainly found in the stratum granulosum [28]. As HSPG was supposed to modulate proliferation and differentiation by its ability to affect growth factor signaling and binding, heparanase could play an important role in keratinocyte differentiation by acting on heparan sulfate. The inner root sheath of hair follicle is also keratinized and its keratinocytes terminal differentiation process could share some traits with epidermal terminal differentiation process [26–28]. In our study, heparanase localization was observed in the basal cells of the oral and nasal epithelium at P0; these findings indicated that heparanase could have contributed to regeneration of and cell renewal in the epithelium. In addition, some epithelial cells and underlying mesenchymal cells facing the palatal shelves at the bottom of tongue at E13.5 and the palatal mesenchyme at E14.5 showed heparanase reactivity, suggesting that heparanase could be involved in the generation of these cells, similar to in the hair cycle and in epidermal physiology. One might speculate that heparanase activity might play important role in the migration and remodeling of the palatal mesenchyme during palate formation.

The appearance of heparanase labeling in the cytoplasm seemed to coincide with lysosomal localization of the enzyme. Our results are also consistent with previous findings that heparanase localized to lysosomes in fibroblasts, osteoblasts, osteo (chondro) clasts, and tumor cells [22, 23, 29, 30]. Specific localization of the latent and active heparanase forms has been detected to perinuclear vesicles, suggesting that heparanase processing and activation occurs in lysosomes [29, 30]. In other cases, heparanase appeared less localized and more diffusely distributed in the cytoplasm, suggesting that under different biological situations, heparanase may be localized in different cellular compartments and hence may exert diverse functions [29]. The determination of its exact role requires further investigation, including electron microscopy studies to examine its exact location.

Heparanase, an endo-*β*-D-glucuronidase expressed in a variety of tissues and cells during normal development and in pathological conditions, can selectively cleave perlecan and syndecan, and this enzyme releases complexes of HS fragments that are bound to the core protein [31]. These released HS complexes, such as growth factors, promote cell growth and migration [32]. Moreover, recent studies indicate that HSPG-growth factor complexes become available in bioactive form for binding to the cognate receptors to promote growth factor-mediated signaling [33–35]. Therefore, it is possible that heparanase-labeled cells contribute to degradation of HS chains present in the palate basement membrane and that release of growth factors might accelerate the proliferation and differentiation of mesenchymal cells of the palatal shelves. In addition to heparanase localization, cells of the MES had MMP-9, MMP-2, and MMP-3 reactivity. However, there were some differences in the distributions of these proteins. For example, MMP-9 was apparently co-localized with the basement membrane, and moderate MMP-2 and intense MMP-3 labeling were seen in the MES cells. Additionally, these MMPs were evident in mesenchyme around the MES. MMPs are involved in the degradation of ECM during normal physiological processes, such as embryonic development, reproduction, and tissue remodeling; MMPs also degrade the ECM during disease processes [36]. The localization of heparanase and MMPs in this study suggests that they have the ability to participate in ECM remodeling during palate growth and formation. MMP-9, MMP-2, and MMP-3 may be involved in the degradation of basement membrane proteins, including type IV collagen, laminin, and perlecan. In our perlecan double-labeling experiments, the basement membrane of the MES had faint perlecan labeling even though laminin was present. In addition, the basement membrane of the MES contained type IV collagen though perlecan was absent; however, we were unable to detect any differences between heparan sulfate and perlecan localization in the basement membrane of the MES. Based on these results, it is conceivable that the degradation of components of the palatal basement membrane may occur in a regulated sequence. Thus, given the localization of heparanase, we propose that the degradation of the MES basement membrane results from the coordinated activity of several proteolytic enzymes. This is supported by a previous study demonstrating that heparanase knockout mice developed normally, were fertile, and exhibit no apparent anatomical or functional abnormalities despite the complete lack of heparanase gene expression and enzymatic activity [37]. Heparanase deficiency was compensated by a tissue-specific marked elevation of MMP family members such as MMP-2, MMP-9, and MMP-14 [37]. These findings provided the evidence for cooperation between heparanase and MMPs in spite of their different enzymatic substrate and suggested that a combined interdependent control mechanism between heparanase and MMPs regulates the ECM degrading enzyme in cells and tissues.

Three processes—programmed cell death [38, 39], cell migration to the oral and nasal side of the palate [40], and epithelial-mesenchymal transdifferentiation (EMT) [41]—have been proposed as the mechanisms involved in the disappearance of the MES. Here, nucleus condensation, a change characteristic of programmed cell death, was evident during histological examination in some MES cells. Additionally, MMP-3, which is known to induce EMT in mammary epithelial cells [42], localized in the MES cells, confirming the possibility that EMT occurred in the MES cells [42]. However, based on our data, we could not determine whether the MES cells underwent, separately or concurrently, any of these three processes. Further studies are required to understand the precise mechanism by which the MES cells vanish from palate.

## 5. Conclusions

In conclusion, we provided evidence that heparanase localized in the palate epithelial cells, the MES cells, and osteoblasts during palate formation, whereas signal from the components of the basement membrane was faint and weak in the palatal shelves. We also observed MMP-9, MMP-2, and MMP-3 signals in the MES, while perlecan, laminin, and collagen type IV signals disappeared from the basement membrane of the MES. Hence, the distribution heparanase and MMPs were consistent with the hypothesis that these proteins had a role in palate ECM remodeling; additionally, these findings suggest that heparanase, in cooperation with other proteases, contributed to palate development. Our results also indicated that further analysis of heparanase might shed light on the fate of the MES in palatogenesis.

## Supplementary Material

Figure S1: Light micrograph showing the negative control, in which the primary antibody was omitted in the MES of palate of a mouse embryo at E15.5. No labeling was observed. Adjacent section to Figure 1F and Figure 4A. O, oropharynx; N, nasopharynx. Bar: 25 *µ*m.
