# Supplementary material for: Heparanase Localization during Palatogenesis in Mice
Source: Biomed Res Int. 2013 Feb 12;2013:760236. doi: 10.1155/2013/760236 (PMC3583076; doi:10.1155/2013/760236)
Supplement: Supplementary file 1 — Figure S1: Light micrograph showing the negative control, in which the primary antibody was omitted in the MES of palate of a mouse embryo at E15.5. No labeling was observed. Adjacent section to Figure 1F and Figure 4A. O, oropharynx; N, nasopharynx. Bar: 25 µm. [file 760236.f1.pdf]

Supplemental data

Figure legend

Figure S1: Negative control, in which the primary antibody was omitted, is shown. Adjacent section to Figure 1F and Figure 4A. O, oropharynx; N; nasopharynx. Bars: 25 μm.

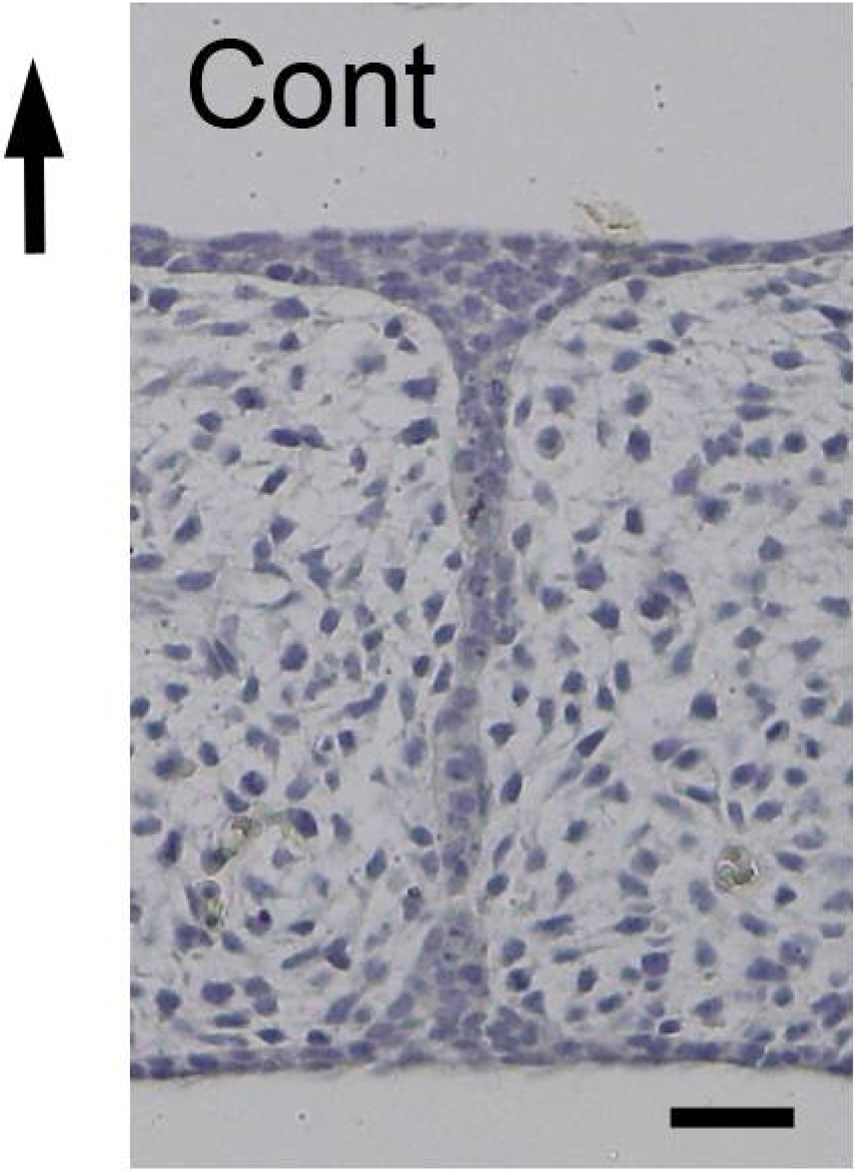

Figure S1. Hirata, A., et al.
